# A three-component, Zn(OTf)_2_-mediated entry into trisubstituted 2-aminoimidazoles

**DOI:** 10.3762/bjoc.15.103

**Published:** 2019-05-07

**Authors:** Alexei Lukin, Anna Bakholdina, Anna Kryukova, Alexander Sapegin, Mikhail Krasavin

**Affiliations:** 1 Lomonosov Institute of Fine Chemical Technologies, MIREA – Russian Technological University, Moscow 119571, Russian Federation; 2 Saint Petersburg State University, Saint Petersburg 199034, Russian Federation

**Keywords:** alkyne hydroamination, cyclocondensation, Lewis acid catalysis, multicomponent reactions, propargylurea

## Abstract

A three-component reaction involving in situ generation of propargylureas and subsequent Zn(OTf)_2_-mediated cyclocondensation with a primary amine yielded trisubstituted 2-aminoimidazoles. These findings are in contrast to the previously reported base-promoted unimolecular cyclization of propargylureas (leading to 2-imidazolones) and extend the range of Lewis acid-catalyzed azole syntheses based on *N*-carbonyl propargylamines.

## Introduction

**Figure 1:**
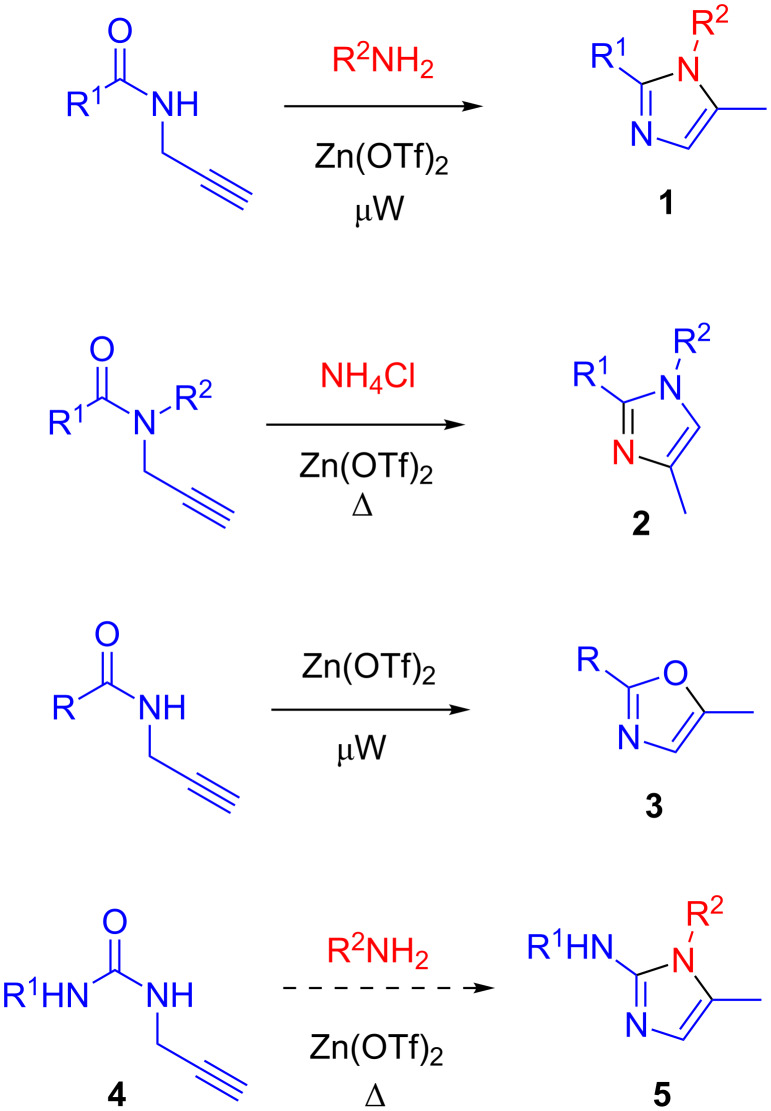
Examples of imidazole (**1** and **2**) and oxazole (**3**) syntheses from propargylamides previously reported and the reaction of propargylureas **4** with primary amines studied in this work.

The pioneering publications of Beller and co-workers describing Zn(OTf)_2_-catalyzed, microwave-promoted conversion of a mixture of a secondary propargylamide and an amine into a trisubstituted imidazole **1** [1–2] inspired us to explore several variants of this methodology. Last year, we described the synthesis of differently substituted imidazoles **2** from tertiary propargylamides and ammonium chloride under conventional heating [3]. More recently, we applied the Beller protocol to the synthesis of 2-substituted 5-methyloxazoles **3** from secondary propargylamides [4]. Being curious to explore more variants of *N*-carbonyl propargylamines, we turned our attention to propargylureas **4**. These have been previously converted to the respective 2-imidazolones via base-promoted intramolecular amination of the propargyl group [5–6]. However, such transformations have not been studied under transition metal or Lewis acid catalysis. Moreover, the possibility to incorporate of an external primary amine into the cyclization process, which would lead to trisubstituted 2-aminoimidazoles **5** has not been explored (Figure 1). 2-Aminoimidazoles have a remarkably broad utility in medicinal chemistry [7]. Moreover, ureas **4** can, in principle, be generated in situ from the respective isocyanates and propargylamine (or propagyl isocyanate and primary amines), thus could offer an opportunity to synthesize 2-aminoimidazoles **5** in a three-component format. Considering these premises, we started to explore the Zn(OTf)_2_-catalyzed reaction of **4** with primary amines. Herein, we present the preliminary results of these studies.

## Results and Discussion

Urea **4a** (prepared by reacting propargylamine with 4-(trifluoromethoxy)benzyl isocyanate) was reacted with an equivalent amount of benzylamine in refluxing toluene in the presence of various Lewis acids. To our delight, the desired product **5a** was observed and isolated in all cases involving Zn(OTf)_2_ catalysis. However, in contrast to previous reports on the preparation of related compounds [1–4], the reaction required substantial amounts (optimally, 50 mol %) of Zn(OTf)_2_ to achieve the best yield (76%). Notably, neither Sc(OTf)_2_ nor Cu(OTf)_2_ employed as catalysts produced a trace of the desired product. To rule out catalysis by adventitious TfOH, the reaction was performed in the presence of an equimolar amount of triflic acid, but no conversion could be detected (Table 1).

**Table 1:** Catalyst screening results for the conversion of **4a** to **5a**.

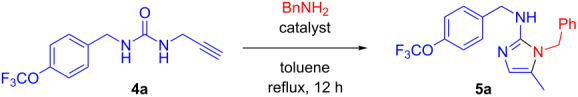		

Entry	Catalyst	Isolated yield (%)

1	Zn(OTf)_2_ (5 mol %)	8
2	Zn(OTf)_2_ (25 mol %)	28
3	Zn(OTf)_2_ (50 mol %)	76
4	Sc(OTf)_2_ (25 mol %)	0
5	Cu(OTf)_2_ (25 mol %)	0
6	TfOH (100 mol %)	0
7	none	0

**Figure 2:**
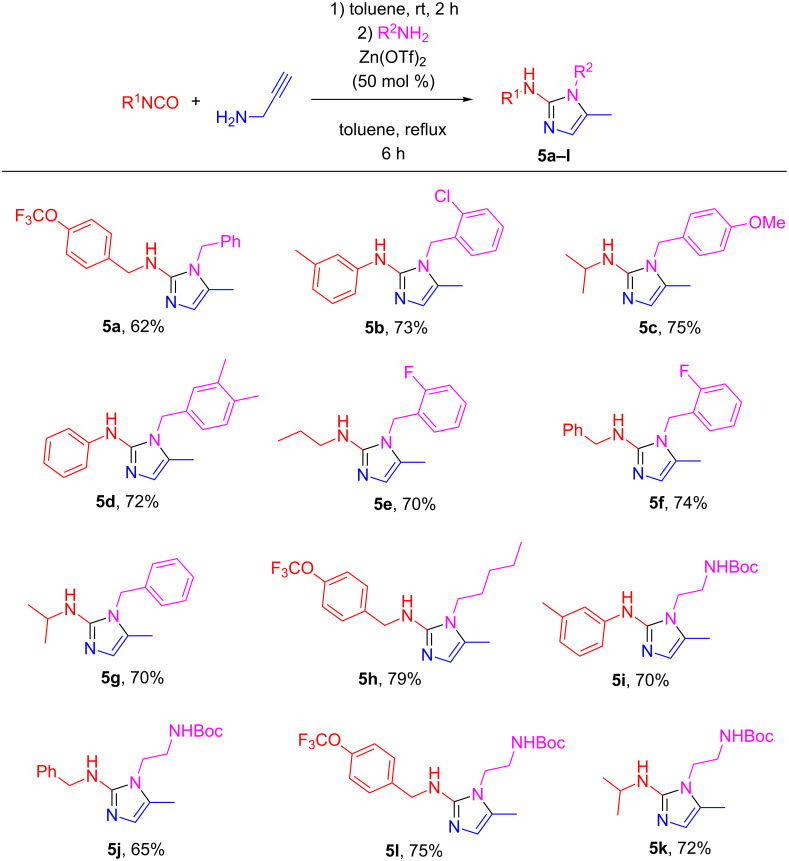
Substrate scope for the three-component synthesis of **5**.

Considering that in situ preparation of ureas **4** could, in principle, enable a three-component entry to imidazoles **5** (an attractive option from the standpoint of library array synthesis), we compared the isolated yield of the above reaction (with the ready-made urea **4a**) with the yield obtained in the three-component format. To our delight, the three-component format led to only a slightly lower yield of **5a** (62%). Viewing this as a worthy toll for the convenience of multicomponent chemistry, we proceeded investigating the scope of the newly established synthesis of trisubstituted 2-aminoimidazoles **5** (Figure 2).

As it is evident from the data presented in Figure 2, the newly developed approach to the synthesis of medicinally important 2-imidazolines allows for independent variation of two periphery elements and provides generally good yields of the target compounds. The reaction can be performed with both, aromatic and aliphatic isocyanates. However, its scope is limited to aliphatic amines as the reaction failed to work for anilines (even electron-rich ones, such as *p*-anisidine). The tolerance of acid-labile protecting groups such as Boc is particularly useful as it offers an opportunity for further side-chain modifications.

**Figure 3:**
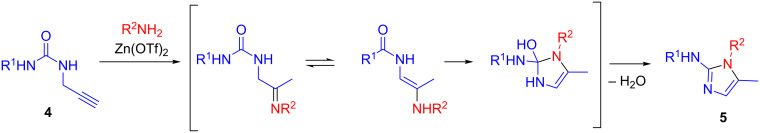
Plausible mechanism for the formation of **5**.

From the mechanistic prospective, the reaction probably proceeds via Zn(OTf)_2_-catalyzed alkyne hydroamination followed by cyclodehydration as depicted in Figure 3, in full analogy with the previously proposed mechanism [2–3].

## Conclusion

In summary, we have successfully employed propargylureas in the synthesis of trisubstituted 2-aminoimidazoles. This is the first example illustrating the utility of such ureas in the synthesis of imidazoles as previously reported syntheses only involved base-promoted cyclization into 2-imidazolones. The reaction can be conveniently conducted in a three-component format which makes it a useful tool for library array synthesis. The investigation of other metal-catalyzed transformations of propargylureas is underway in our laboratories and will be reported in due course.

## Supporting Information

File 1 General experimental information, synthetic procedures, analytical data and NMR spectra for the reported compounds.
