# Supplementary material for: A three-component, Zn(OTf)2-mediated entry into trisubstituted 2-aminoimidazoles
Source: Beilstein J Org Chem. 2019 May 7;15:1061–4. doi: 10.3762/bjoc.15.103 (PMC6541334; doi:10.3762/bjoc.15.103)

BEILSTEIN JOURNAL OF ORGANIC CHEMISTRY

Supporting Information

for

A three-component, $Zn(OTf)_2$-mediated entry into trisubstituted 2-aminoimidazoles

Alexei Lukin, Anna Bakholdina, Anna Kryukova, Alexander Sapegin and Mikhail Krasavin

Beilstein J. Org. Chem. **2019,** *15,* 1061–1064. doi:10.3762/bjoc.15.103

General experimental information, synthetic procedures, analytical data and NMR spectra for the reported compounds

License and Terms: This is a supporting information file under the terms of the Creative Commons Attribution License (http://creativecommons.org/licenses/by/4.0). Please note that the reuse, redistribution and reproduction in particular requires that the authors and source are credited.
The license is subject to the *Beilstein Journal of Organic Chemistry* terms and conditions: (https://www.beilstein-journals.org/bjoc)

Table of Contents:

Experimental procedures S2–S5

Copies of ^{1}H and ^{13}C NMR spectra of compounds **5a–l** S6–S17

Experimental procedures

General

All reactions were conducted in oven-dried glassware in an atmosphere of nitrogen. Melting points were measured with a Büchi B-520 melting point apparatus and were not corrected. Analytical thin-layer chromatography was carried out on Silufol UV-254 silica gel plates using appropriate mixtures of ethyl acetate and hexane. Compounds were visualized with short-wavelength UV light. ^{1}H NMR and ^{13}C NMR spectra were recorded on a Bruker MSL-300 spectrometer in DMSO-d_6 using TMS as an internal standard. Mass spectra were recorded using a Shimadzu LCMS-2020 system with electron impact (EI) ionization. All and reagents and solvents were obtained from commercial sources and were used without purification. Compounds **5b**–**l** were synthesized according to the procedure described for the preparation of compound **5a**.

1-Benzyl-5-methyl-*N*-[4-(trifluoromethoxy)benzyl]-1*H*-imidazol-2-amine (5a)

Propargylamine (0.162 mL, 3.0 mmol) was dissolved in anhydrous toluene (10 mL) and treated with a solution of 1-(isocyanatomethyl)-4-(trifluoromethoxy)benzene (0.65 g, 3.0 mmol) in anhydrous toluene (5 mL). The mixture was stirred at rt for 2 h. Benzylamine (0.1 g, 1 mmol) and zinc trifluoromethanesulfonate (0.181 g, 0.5 mmol) were added and the reaction mixture was heated at reflux for 6 hours. Upon cooling to rt, the reaction mixture was washed with 5% aq. K_2CO_3 (2 × 10 mL), dried over Na_2SO_4 and concentrated in vacuo. The residue was purified by column chromatography on silica gel using 0→10% methanol in chloroform as eluent.

Yield 0.22 g (62%), brown oil. ^{1}H NMR (300 MHz, DMSO) δ 7.39 (d, *J* = 8.6 Hz, 2H), 7.32 (t, *J* = 7.1 Hz, 2H), 7.25 (t, *J* = 7.3 Hz, 3H), 7.04 (d, *J* = 7.1 Hz, 2H), 6.30 (t, *J* = 5.7 Hz, 1H), 6.21 (d, *J* = 4.4 Hz, 1H), 5.03 (d, *J* = 11.5 Hz, 2H), 4.38 (t, *J* = 5.7 Hz, 2H), 1.94 (d, *J* = 3.4 Hz, 3H); ^{13}C NMR (75 MHz, DMSO) δ 149.4, 146.9 (d, *J* = 1.8 Hz), 140.8, 138.0, 129.0, 128.5, 127.0, 126.3, 122.0, 120.8, 120.6, 120.2 (q, *J* = 255.7 Hz), 45.5, 44.3, 9.5; HRMS (ESI) m/z calcd for $C_{19}H_{18}F_3N_3O$ [M+H$^+$] 362.1480 Da, found 362.1475 Da.

1-(2-Chlorobenzyl)-5-methyl-*N*-(3-methylphenyl)-1*H*-imidazol-2-amine (5b)

Yield 0.150 g (73%), brown oil. ^{1}H NMR (300 MHz, $CDCl_3$) δ 7.39 – 7.34 (m, 1H), 7.18 (p, *J* = 7.5 Hz, 3H), 7.08 – 7.01 (m, 1H), 6.71 (s, 1H), 6.69 – 6.65 (m, 2H), 6.59 (d, *J* = 7.2 Hz, 1H), 5.04 (s, 2H), 2.22 (s, 3H), 2.06 (s, 3H); ^{13}C NMR (75 MHz, $CDCl_3$) δ 143.9, 143.8, 139.1, 134.1,

132.1, 129.6, 129.0, 128.9, 127.5, 126.9, 125.5, 123.0, 121.5, 116.4, 112.9, 44.0, 21.6, 10.0; HRMS (ESI) m/z calcd for $C_{18}H_{19}ClN_3$ [M+H$^+$] 312.1268 Da, found 312.1262 Da.

***N*-Isopropyl-1-(4-methoxybenzyl)-5-methyl-1*H*-imidazol-2-amine (5c)**

Yield 0.142 g (75%), brown oil. ^{1}H NMR (300 MHz, $CDCl_3$) δ 6.96 (d, *J* = 8.7 Hz, 2H), 6.86 – 6.82 (m, 2H), 6.45 (s, 1H), 4.77 (s, 2H), 3.87 – 3.78 (m, 1H), 3.77 (s, 3H), 2.06 (s, 3H), 1.09 (d, *J* = 6.4 Hz, 6H); ^{13}C NMR (75 MHz, $CDCl_3$) δ 159.2, 148.7, 128.3, 127.4, 123.0, 120.2, 114.4, 55.4, 45.5, 44.9, 23.4, 9.9; HRMS (ESI) m/z calcd for $C_{15}H_{21}N_3O$ [M+H$^+$] 260.1763 Da, found 260.1757 Da.

1-(3,4-Dimethylbenzyl)-5-methyl-*N*-phenyl-1*H*-imidazol-2-amine (5d)

Yield 0.121 g (72%), brown oil. ^{1}H NMR (300 MHz, $CDCl_3$) δ 7.17 (dd, *J* = 8.3, 7.5 Hz, 2H), 7.06 (d, *J* = 7.7 Hz, 1H), 6.89 – 6.83 (m, 3H), 6.80 – 6.72 (m, 2H), 6.66 (s, 1H), 4.90 (s, 2H), 2.23 (s, *J* = 9.0 Hz, 3H), 2.20 (s, 3H), 2.12 (s, 3H); ^{13}C NMR (75 MHz, $CDCl_3$) δ 143.9, 143.4, 137.4, 136.2, 133.8, 130.2, 129.2, 127.6, 125.4, 123.7, 122.6, 120.4, 115.7, 46.1, 19.8, 19.4, 10.2; HRMS (ESI) m/z calcd for $C_{19}H_{22}N_3$ [M+H$^+$] 292.1814 Da, found 292.1808 Da.

1-(2-Fluorobenzyl)-5-methyl-*N*-propyl-1*H*-imidazol-2-amine (5e)

Yield 0.139 g (70%), brown oil. ^{1}H NMR (300 MHz, $CDCl_3$) δ 7.42 (t, *J* = 7.3 Hz, 1H), 7.25 – 7.19 (m, 1H), 7.12 – 6.98 (m, 2H), 6.39 (s, 1H), 4.55 (d, *J* = 5.5 Hz, 2H), 3.89 (br.s, 1H), 3.56 (t, *J* = 7.4 Hz, 2H), 2.10 (s, 3H), 1.70 – 1.56 (m, 2H), 0.91 (t, *J* = 7.4 Hz, 3H); ^{13}C NMR (75 MHz, $CDCl_3$) δ 161.3 (d, *J* = 245.8 Hz), 148.6, 130.46 (d, *J* = 4.6 Hz), 129.1 (d, *J* = 8.2 Hz), 126.5 (d, *J* = 14.6 Hz), 124.3 (d, *J* = 3.5 Hz), 123.3, 120.5, 115.4 (d, *J* = 21.4 Hz), 42.4 (d, *J* = 3.7 Hz), 23.1, 11.3, 9.9; HRMS (ESI) m/z calcd for $C_{14}H_{19}FN_3$ [M+H$^+$] 248.1563 Da, found 248.1558 Da.

***N*-Benzyl-1-(2-fluorobenzyl)-5-methyl-1*H*-imidazol-2-amine (5f)**

Yield 0.126 g (74%), brown oil. ^{1}H NMR (300 MHz, $CDCl_3$) δ 7.34 – 7.27 (m, 3H), 7.24 – 7.13 (m, 2H), 7.09 – 6.98 (m, 3H), 6.96 – 6.77 (m, 1H), 6.47 (s, 1H), 4.92 (d, *J* = 14.7 Hz, 2H), 4.52 – 4.44 (m, 2H), 2.08 (s, 3H); ^{13}C NMR (75 MHz, $CDCl_3$) δ 161.0 (d, *J* = 246.2 Hz), 148.5, 135.8, 129.9 (d, *J* = 4.3 Hz), 129.8 (d, *J* = 8.1 Hz), 129.2, 128.6, 127.9, 127.4, 126.2, 124.8 (d, *J* = 3.6 Hz), 124.2 (d, *J* = 3.5 Hz), 115.3 (d, *J* = 21.3 Hz), 45.6, 40.0 (d, *J* = 5.0 Hz), 9.9; HRMS (ESI) m/z calcd for $C_{18}H_{18}FN_3$ [M+H$^+$] 296.1563 Da, found 296.1558 Da.

1-Benzyl-*N*-isopropyl-5-methyl-1*H*-imidazol-2-amine (5g)

Yield 0.114 g (70%), brown oil. ^{1}H NMR (300 MHz, $CDCl_3$) δ 7.37 – 7.27 (m, 3H), 7.04 (d, *J* = 6.9 Hz, 2H), 6.49 (s, 1H), 4.91 (s, 2H), 3.96 – 3.80 (m, 1H), 2.06 (s, 3H), 1.11 (d, *J* = 6.3 Hz, 6H); ^{13}C NMR (75 MHz, $CDCl_3$) δ 148.5, 136.3, 129.1, 127.9, 126.2, 123.2, 119.4, 45.7, 45.5, 23.3, 9.9; HRMS (ESI) m/z calcd for $C_{14}H_{19}N_3Na$ [M+Na$^+$] 252.1471 Da, found 252.1471 Da.

5-Methyl-1-pentyl-*N*-[4-(trifluoromethoxy)benzyl]-1*H*-imidazol-2-amine (5h)

Yield 0.100 g (79%), brown oil. ^{1}H NMR (300 MHz, $CDCl_3$) δ 7.41 (d, *J* = 8.5 Hz, 2H), 7.15 (d, *J* = 8.2 Hz, 2H), 6.38 (s, 1H), 4.51 (d, *J* = 4.8 Hz, 2H), 3.62 (t, *J* = 7.4 Hz, 2H), 2.11 (s, *J* = 6.5 Hz, 3H), 1.67 – 1.55 (m, 2H), 1.33 – 1.26 (m, 4H), 0.88 (t, *J* = 6.8 Hz, 3H); ^{13}C NMR (75 MHz, $CDCl_3$) δ 148.5 (q, *J* = 1.8 Hz), 147.9, 138.0, 129.4, 123.6, 121.1, 120.6 (d, *J* = 256.9 Hz), 118.2, 47.1, 42.6, 29.5, 29.0, 22.5, 14.0, 9.9; HRMS (ESI) m/z calcd for $C_{17}H_{23}F_3N_3O$ [M+H$^+$] 342.1793 Da, found 342.1788 Da.

***tert*-Butyl (2-{5-methyl-2-[(3-methylphenyl)amino]-1*H*-imidazol-1-yl}ethyl)carbamate (5i)**

Yield 0.144 g (70%), brown oil. ^{1}H NMR (300 MHz, $CDCl_3$) δ 7.06 (t, *J* = 7.6 Hz, 1H), 6.87 (s, 2H), 6.65 (d, *J* = 7.4 Hz, 1H), 6.55 (s, 1H), 5.38 (t, *J* = 5.4 Hz, 1H), 3.79 (t, *J* = 6.3 Hz, 2H), 3.21 (q, *J* = 6.3 Hz, 2H), 2.25 (s, 3H), 2.13 (s, 3H), 1.43 (s, 9H); ^{13}C NMR (75 MHz, $CDCl_3$) δ 156.5, 143.5, 138.9, 129.0, 124.4, 121.9, 121.2, 116.4, 112.9, 80.0, 42.1, 40.6, 28.4, 21.6, 9.9; HRMS (ESI) m/z calcd for $C_{18}H_{27}N_4O_2$ [M+H$^+$] 331.2134 Da, found 331.2129 Da.

***tert*-Butyl {2-[2-(benzylamino)-5-methyl-1*H*-imidazol-1-yl]ethyl}carbamate (5j)**

Yield 0.134 g (65%), brown oil. ^{1}H NMR (300 MHz, $CDCl_3$) δ 7.39 (d, *J* = 6.9 Hz, 2H), 7.33 – 7.21 (m, 3H), 6.32 (s, 1H), 4.50 (d, *J* = 4.9 Hz, 2H), 3.82 (t, *J* = 6.5 Hz, 2H), 3.30 (q, *J* = 6.4 Hz, 2H), 2.10 (s, 3H), 1.37 (s, 9H); ^{13}C NMR (75 MHz, $CDCl_3$) δ 156.4, 148.5, 138.8, 128.6, 128.0, 127.5, 123.3, 117.3, 80.2, 47.7, 41.9, 40.1, 28.4, 9.7; HRMS (ESI) m/z calcd for $C_{18}H_{27}N_4O_2$ [M+H$^+$] 331.2134 Da, found 331.2129 Da.

***tert*-Butyl {2-[2-(isopropylamino)-5-methyl-1*H*-imidazol-1-yl]ethyl}carbamate (5k)**

Yield 0.096 g (72%), brown oil. ^{1}H NMR (300 MHz, $CDCl_3$) δ 7.77 (d, *J* = 7.3 Hz, 1H), 6.47 (s, 1H), 6.07 (s, 1H), 4.26 – 4.11 (m, 1H), 3.99 (br.s, 2H), 3.38 (d, *J* = 4.0 Hz, 2H), 2.16 (s, 3H), 1.41 – 1.34 (m, 15H); ^{13}C NMR (75 MHz, $CDCl_3$) δ 156.7, 146.0, 123.6, 109.3, 80.0, 46.9, 42.2, 39.1, 28.5, 22.9, 9.5; HRMS (ESI) m/z calcd for $C_{14}H_{27}N_4O_2$ [M+H$^+$] 283.2134 Da, found 283.2152 Da.

***tert*-Butyl [2-(5-methyl-2-{[4-(trifluoromethoxy)benzyl]amino}-1*H*-imidazol-1-yl)ethyl]carbamate (5l)**

Yield 0.116 g (75%), brown oil. ^{1}H NMR (300 MHz, $CDCl_3$) δ 7.40 (d, *J* = 8.4 Hz, 2H), 7.11 (d, *J* = 8.1 Hz, 2H), 6.33 (s, 1H), 4.49 (s, 2H), 3.80 (t, *J* = 6.5 Hz, 2H), 3.26 (q, *J* = 6.2 Hz, 2H), 2.08 (s, 3H), 1.34 (s, 9H); ^{13}C NMR (75 MHz, $CDCl_3$) δ 156.6, 148.5 (q, *J* = 1.8 Hz), 148.1, 137.4, 129.4, 123.4, 121.1, 120.5 (d, *J* = 256.9 Hz), 116.1, 80.3, 46.7, 41.8, 40.0, 28.3, 9.6; HRMS (ESI) m/z calcd for $C_{19}H_{26}F_3N_4O_3$ [M+H^{+}] 415.1957 Da, found Da 415.1952.

^{1}H and ^{13}C NMR spectra for compound **5a**

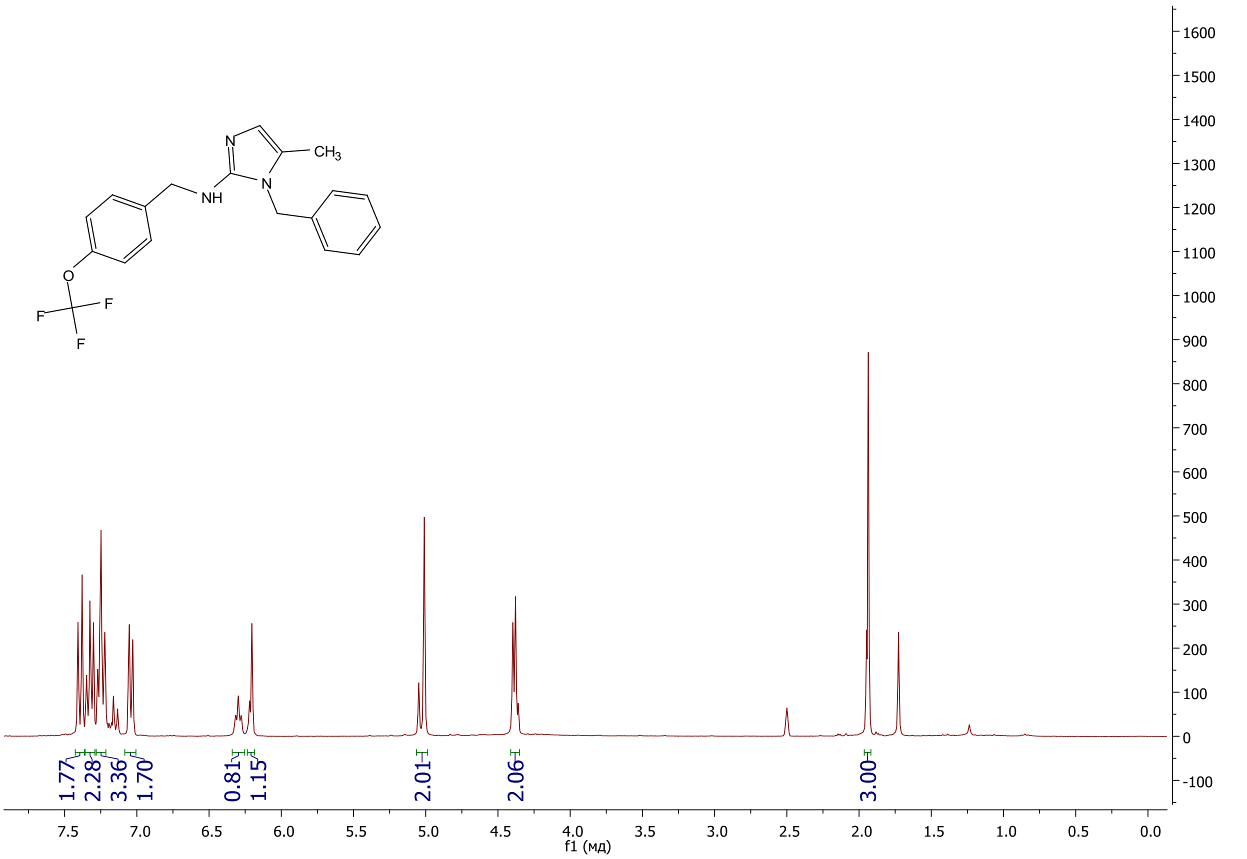

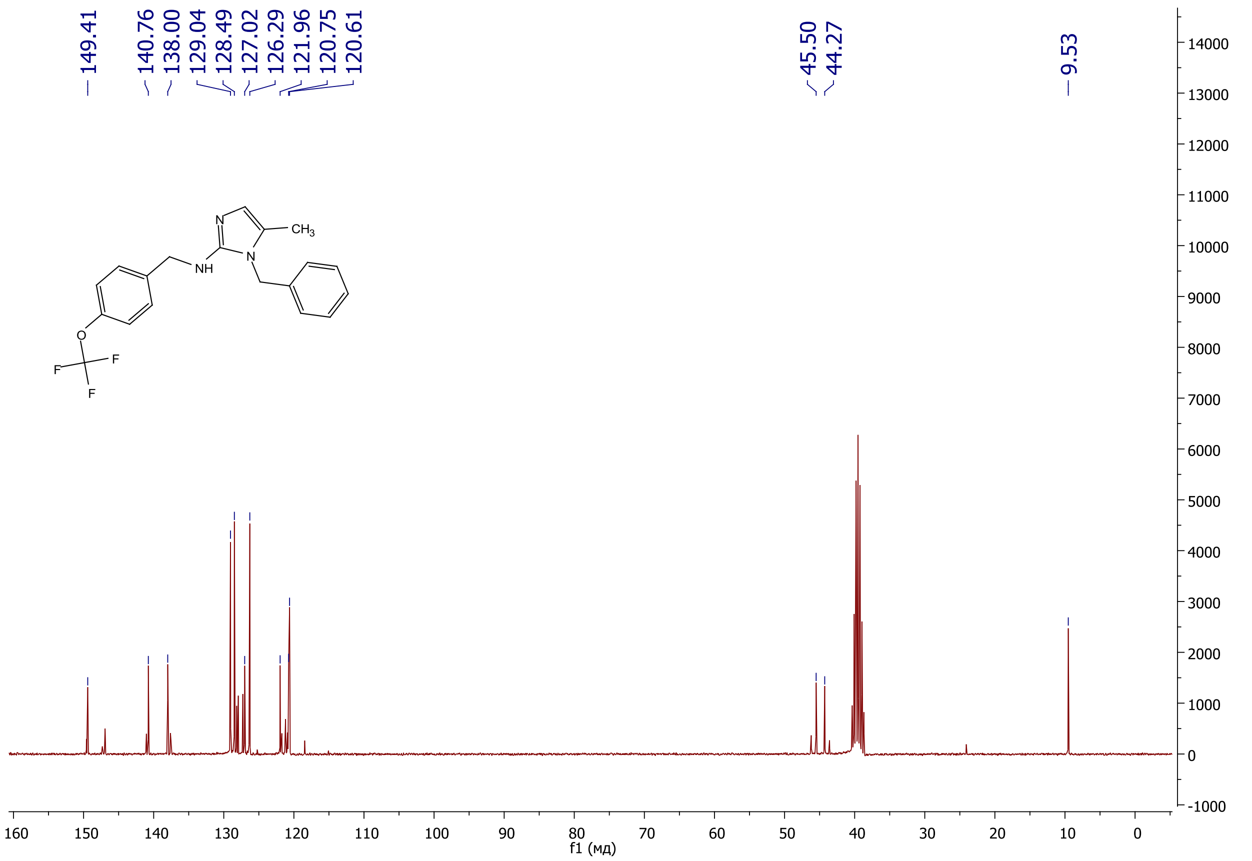

^{1}H and ^{13}C NMR spectra for compound **5b**

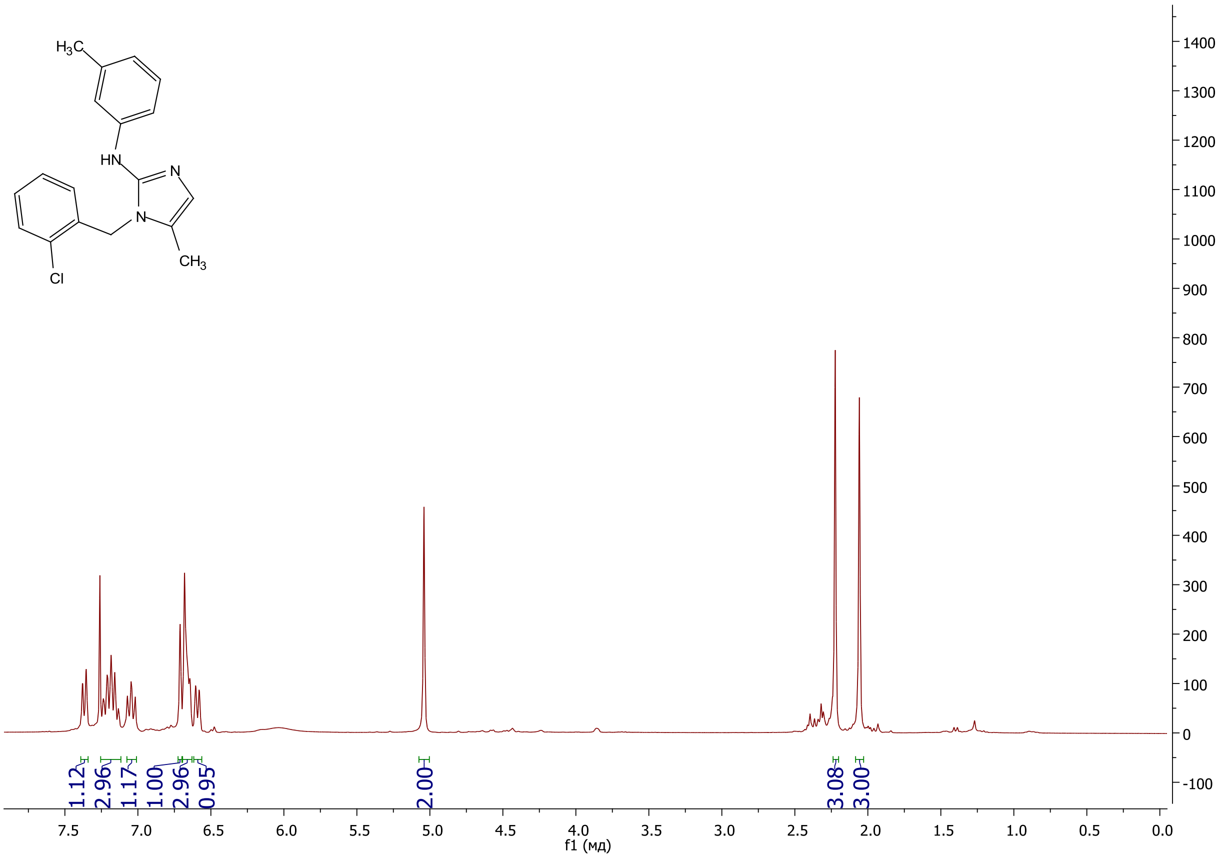

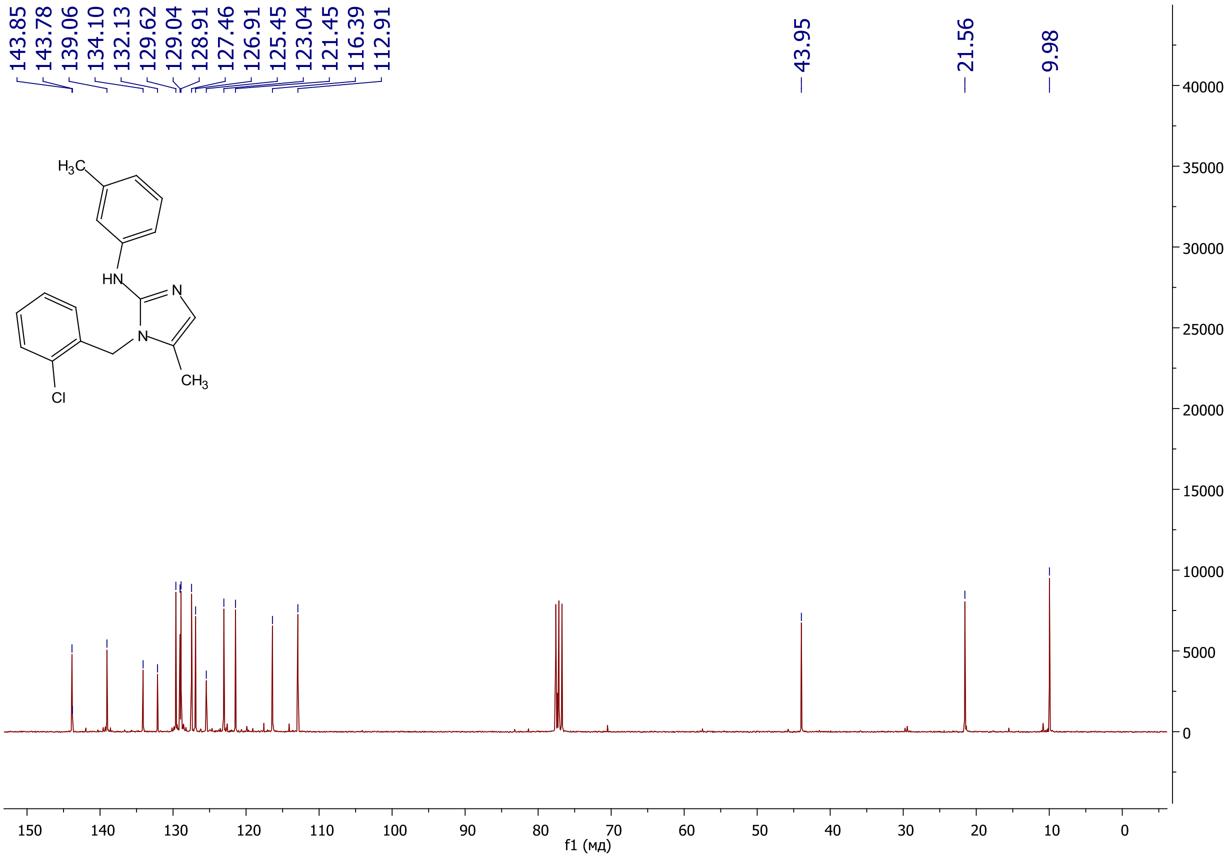

^1H and ^{13}C NMR spectra for compound **5c**

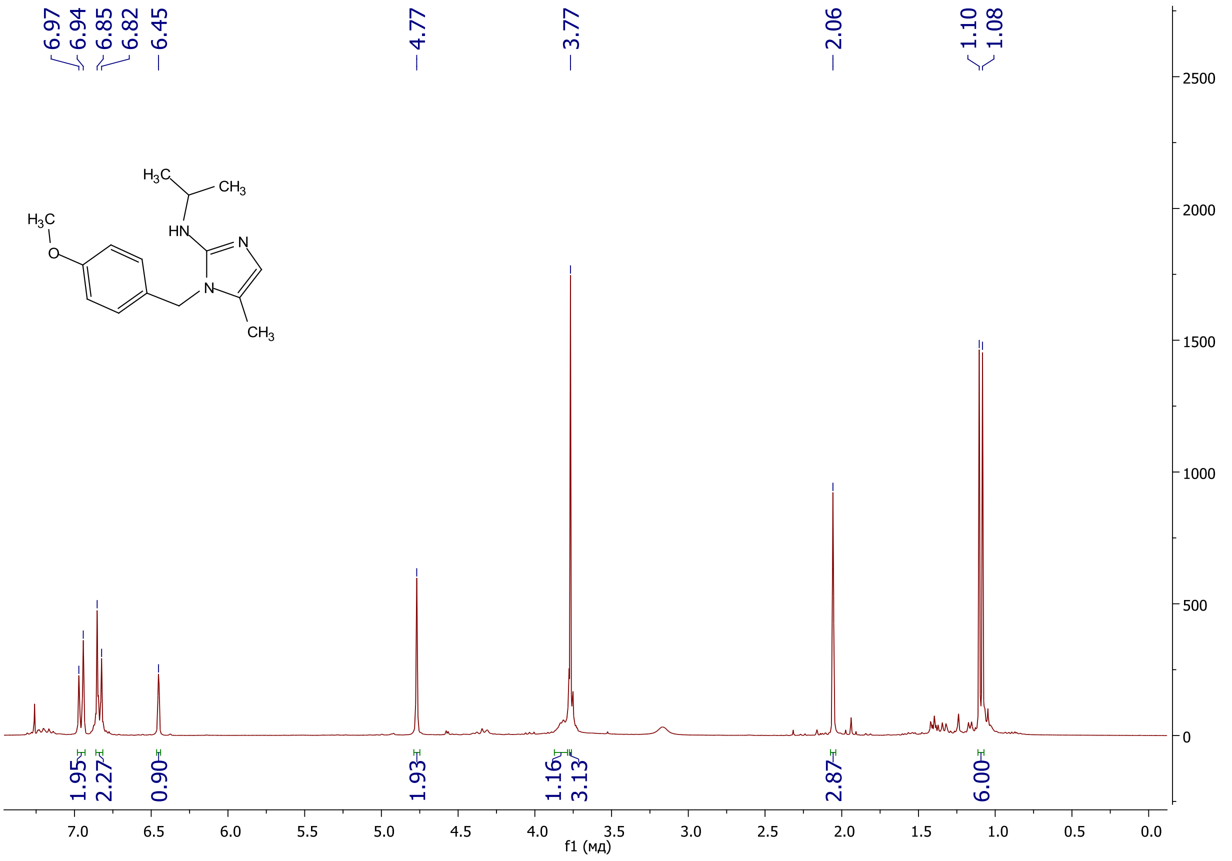

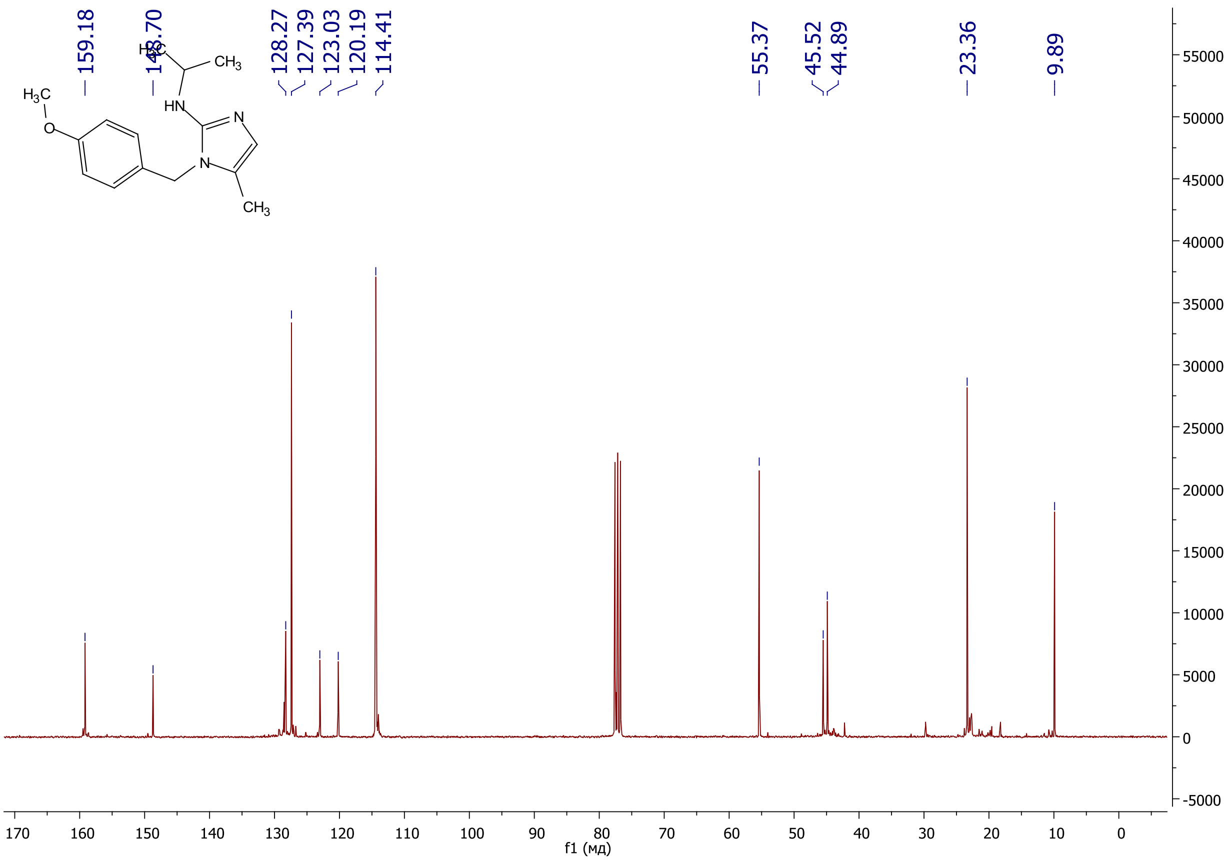

^{1}H and ^{13}C NMR spectra for compound **5d**

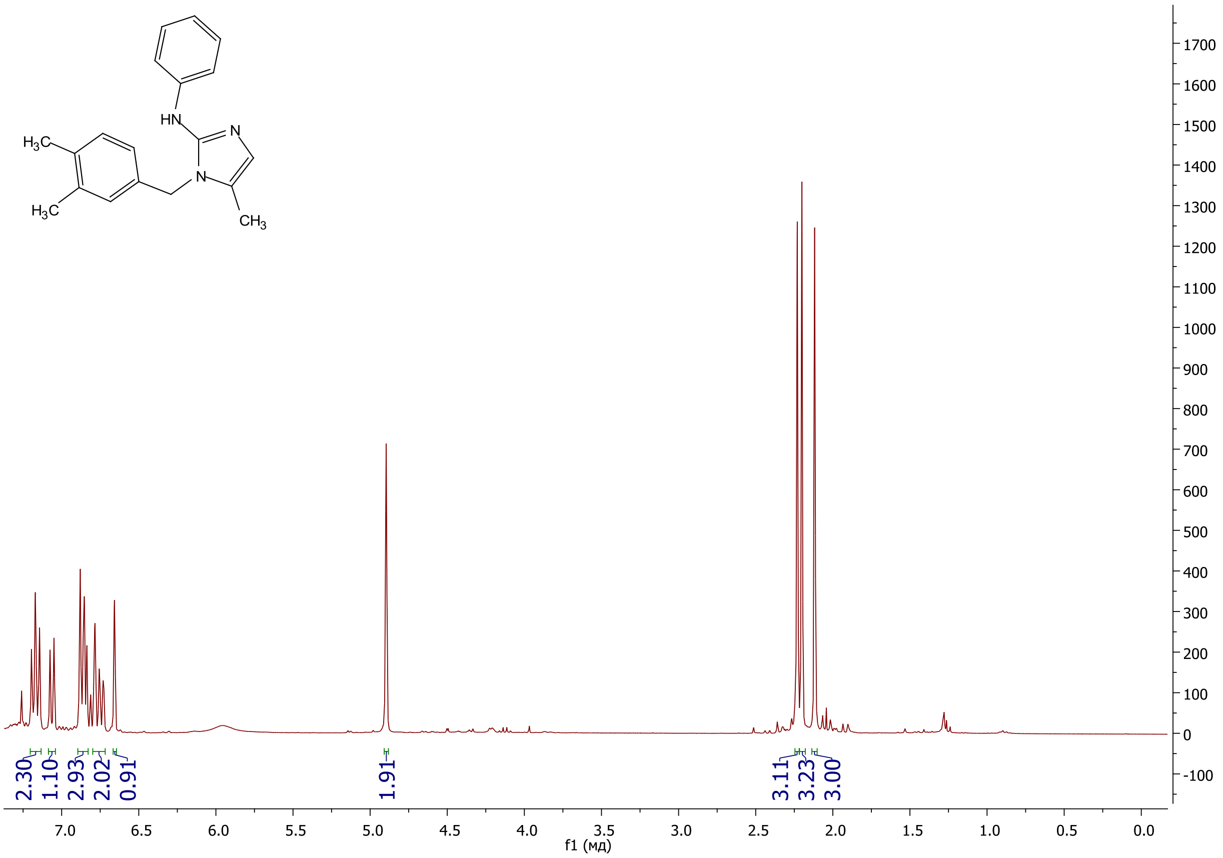

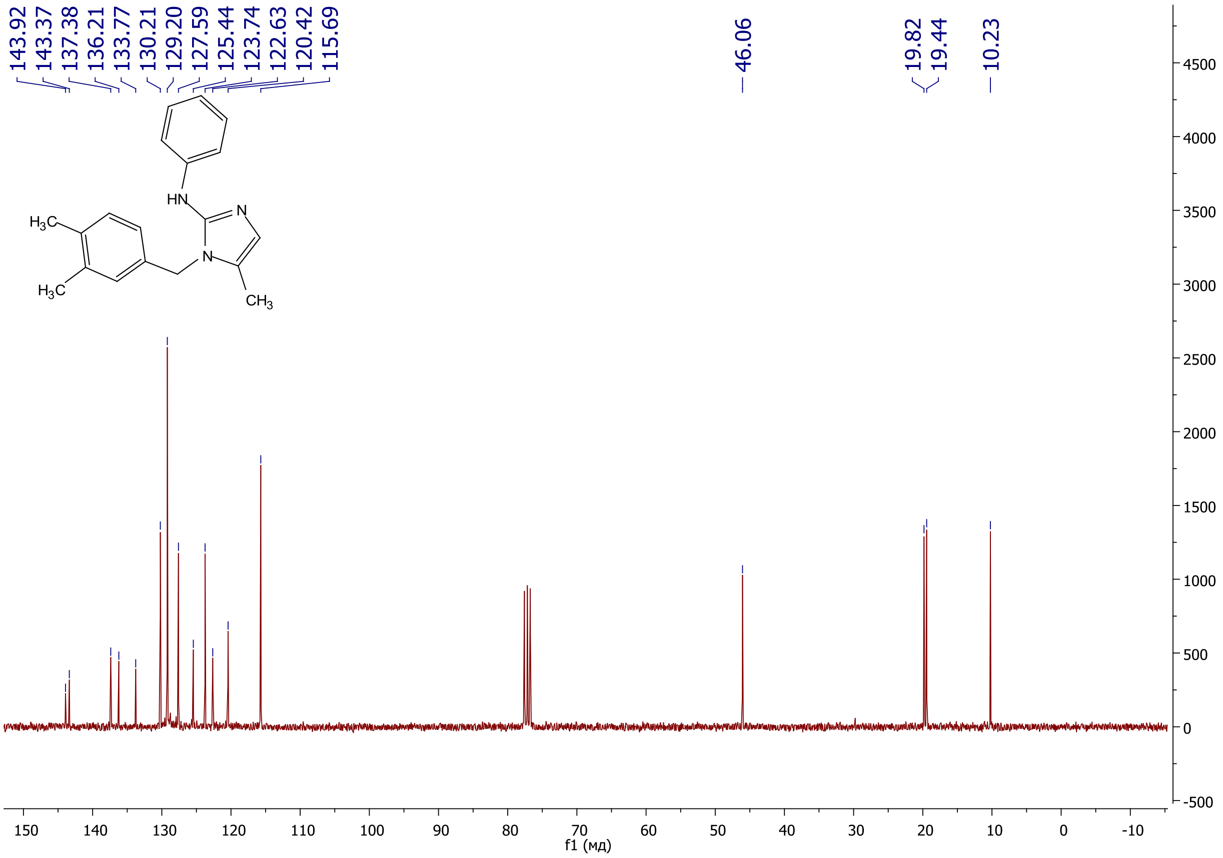

^{1}H and ^{13}C NMR spectra for compound **5e**

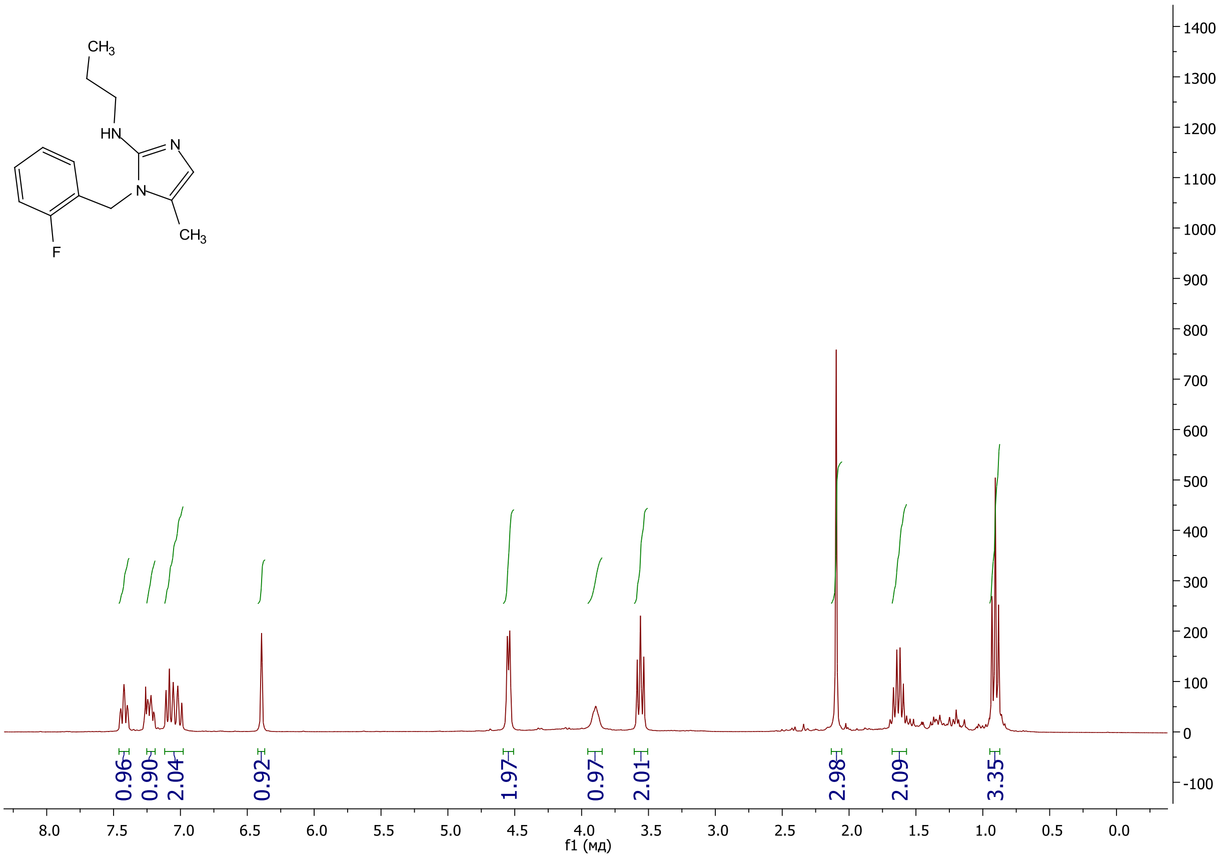

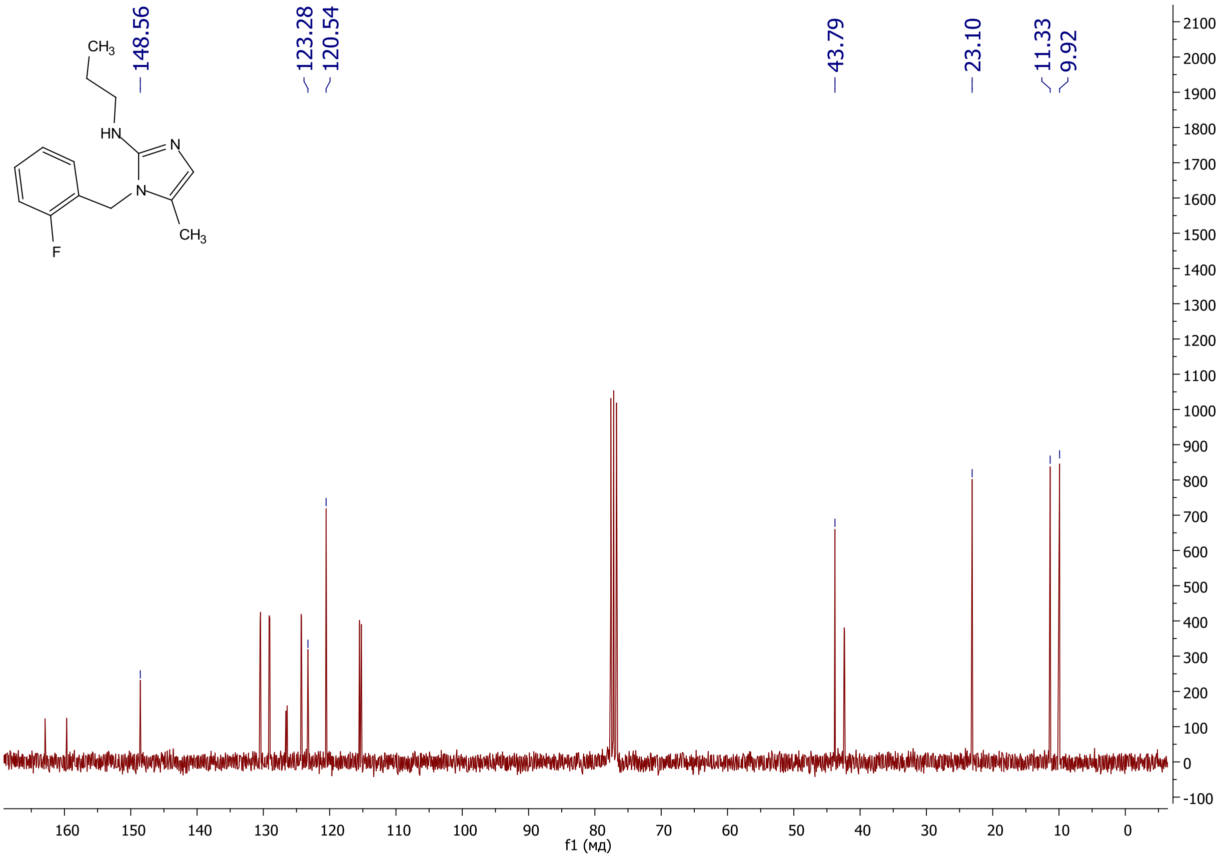

^{1}H and ^{13}C NMR spectra for compound **5f**

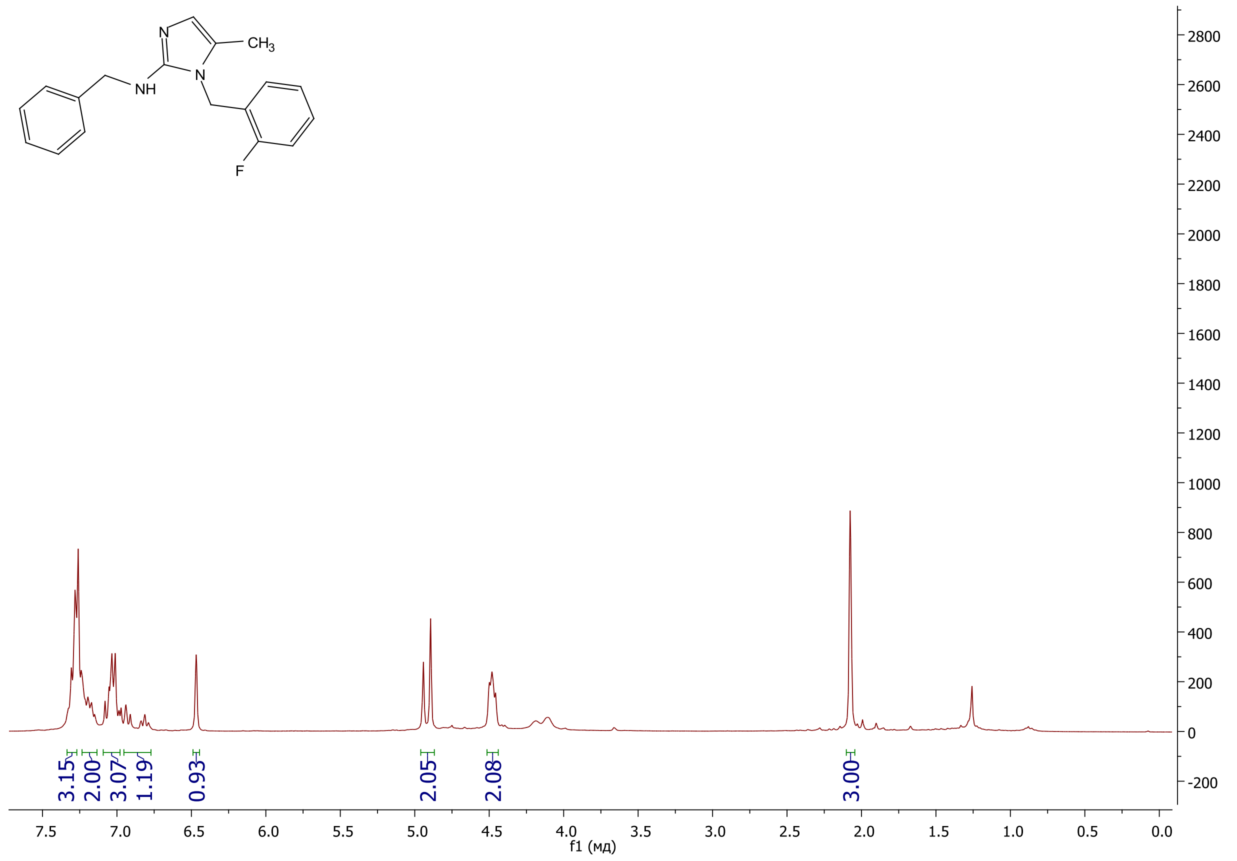

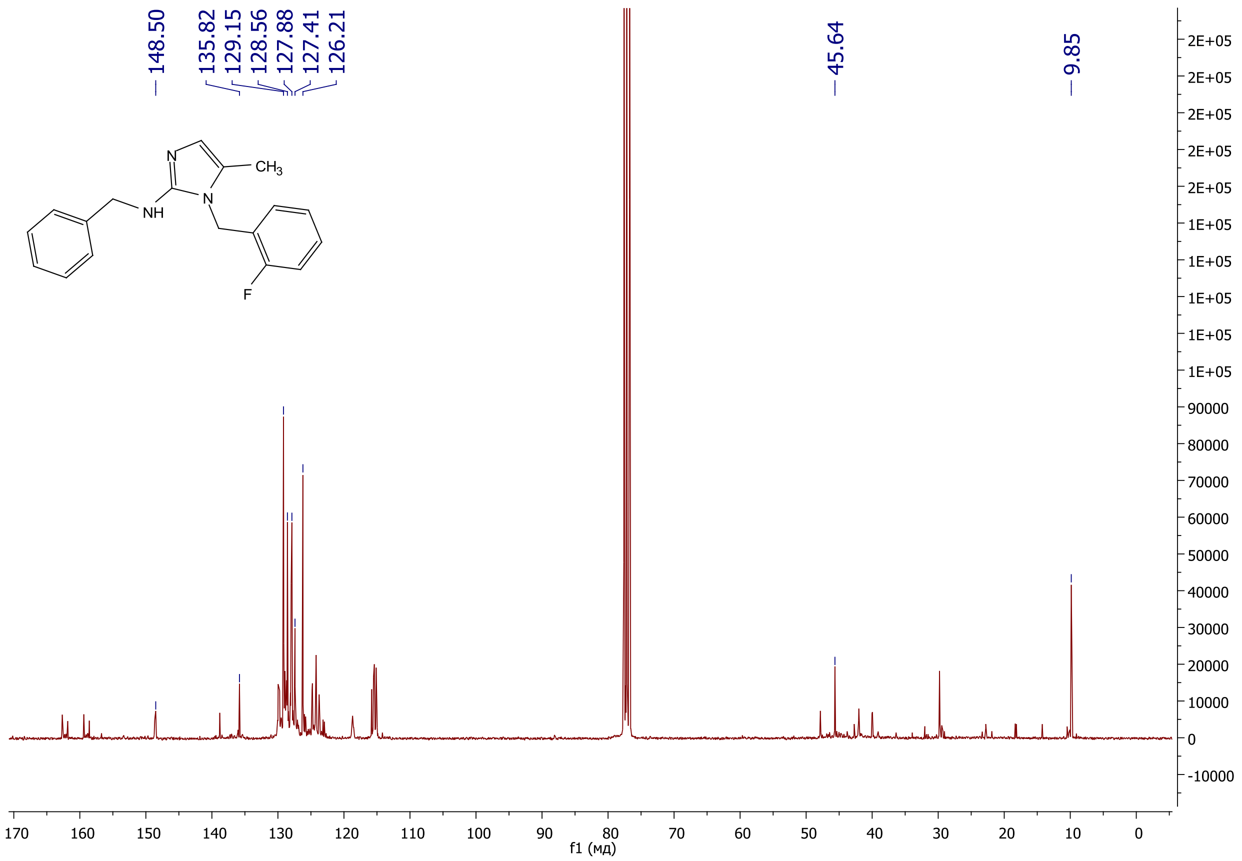

^{1}H and ^{13}C NMR spectra for compound **5g**

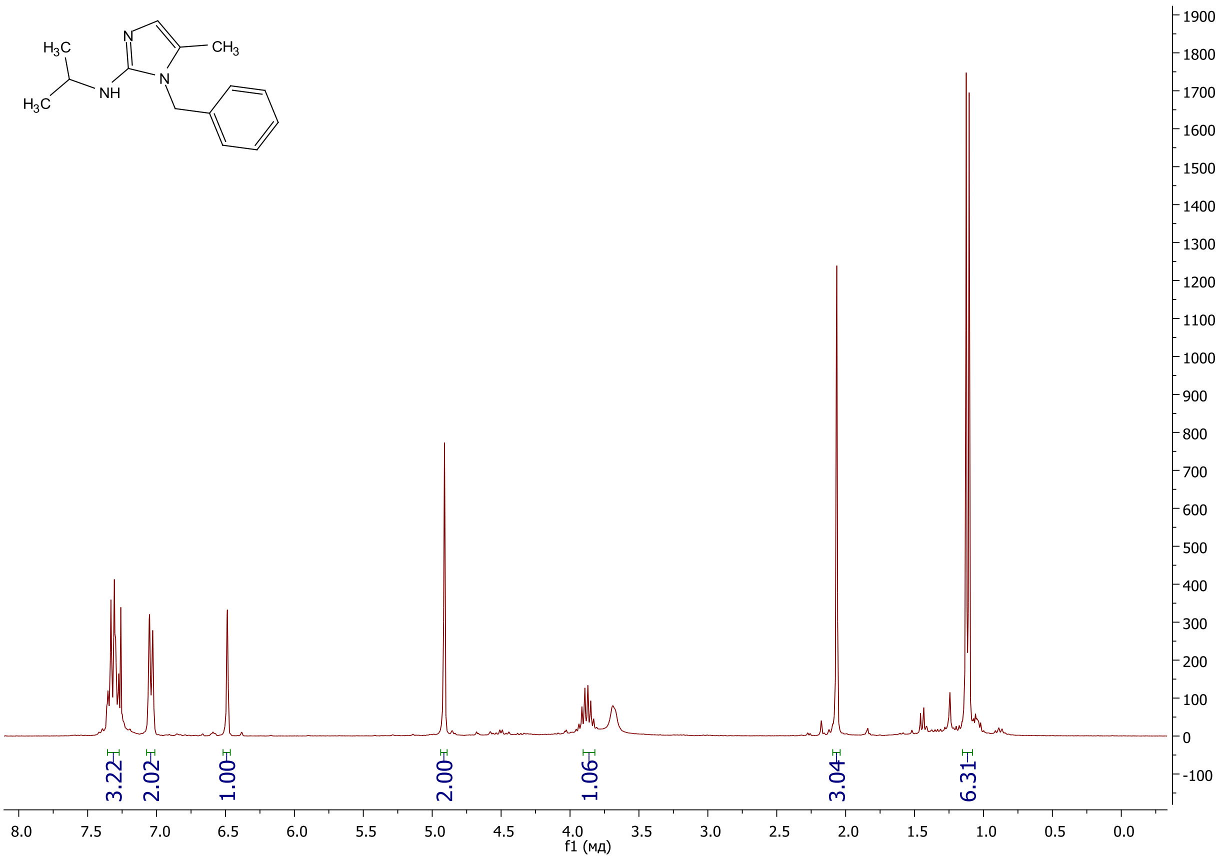

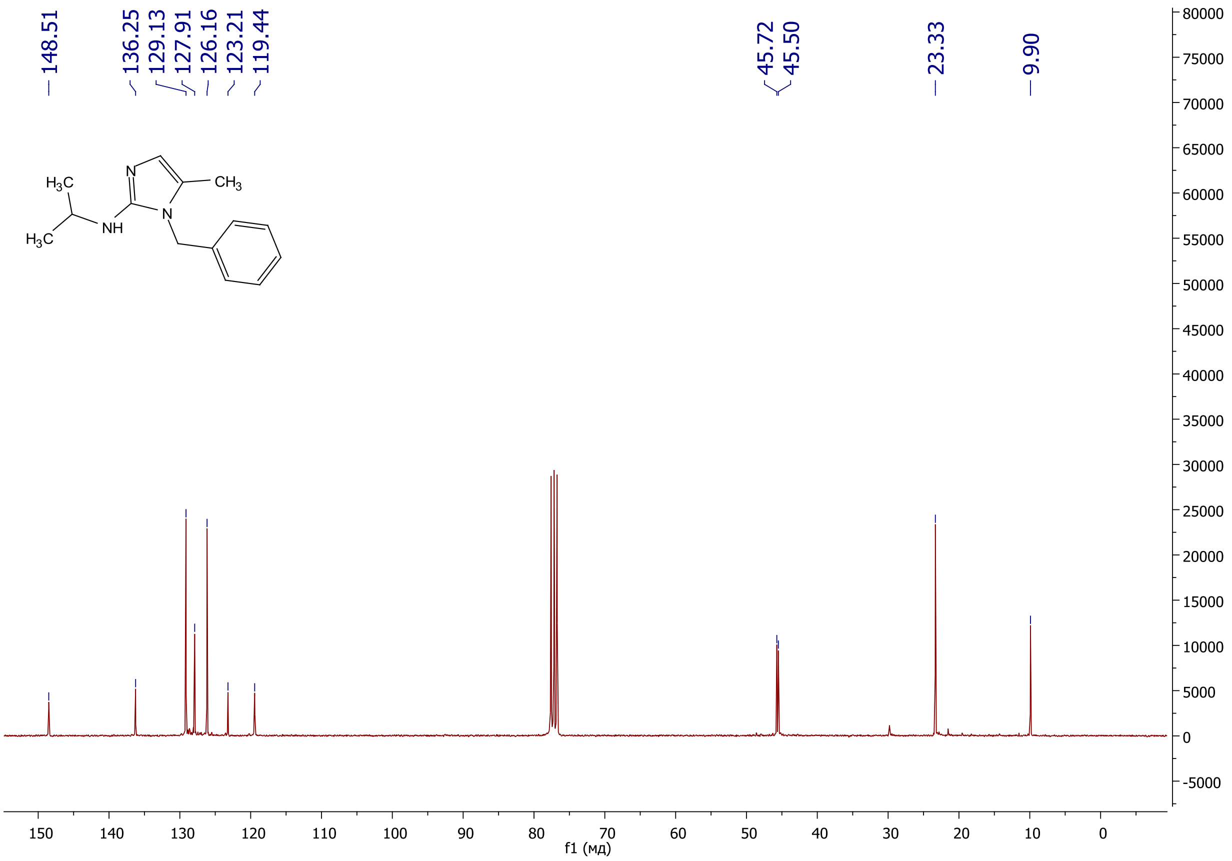

^{1}H and ^{13}C NMR spectra for compound **5h**

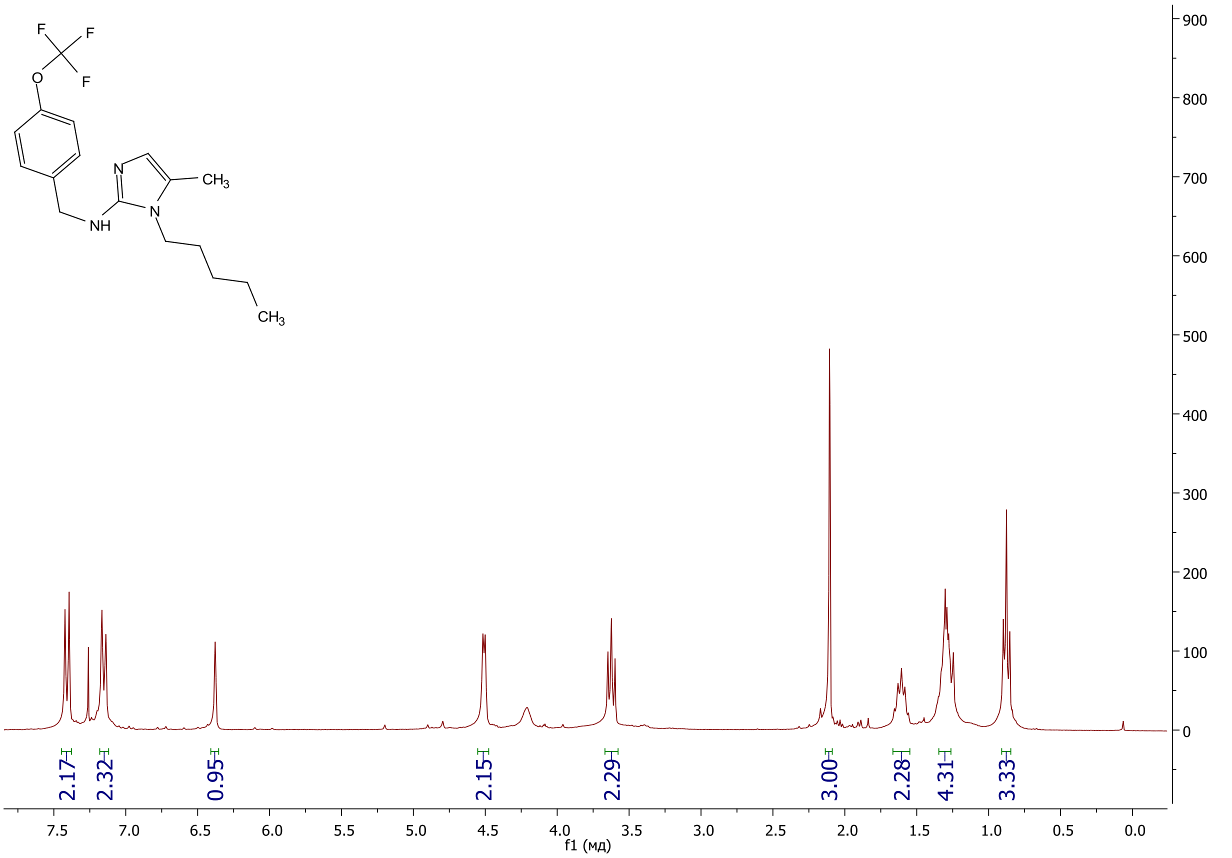

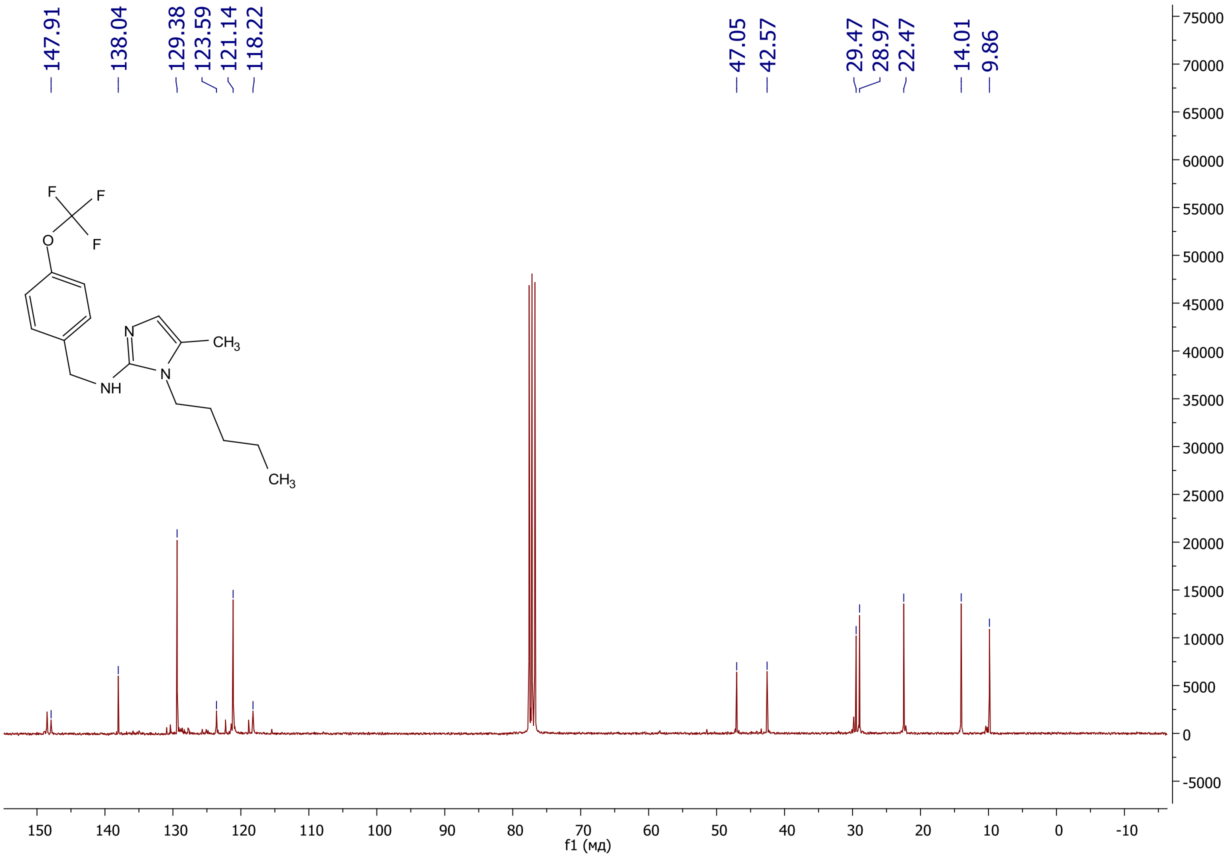

^{1}H and ^{13}C NMR spectra for compound **5i**

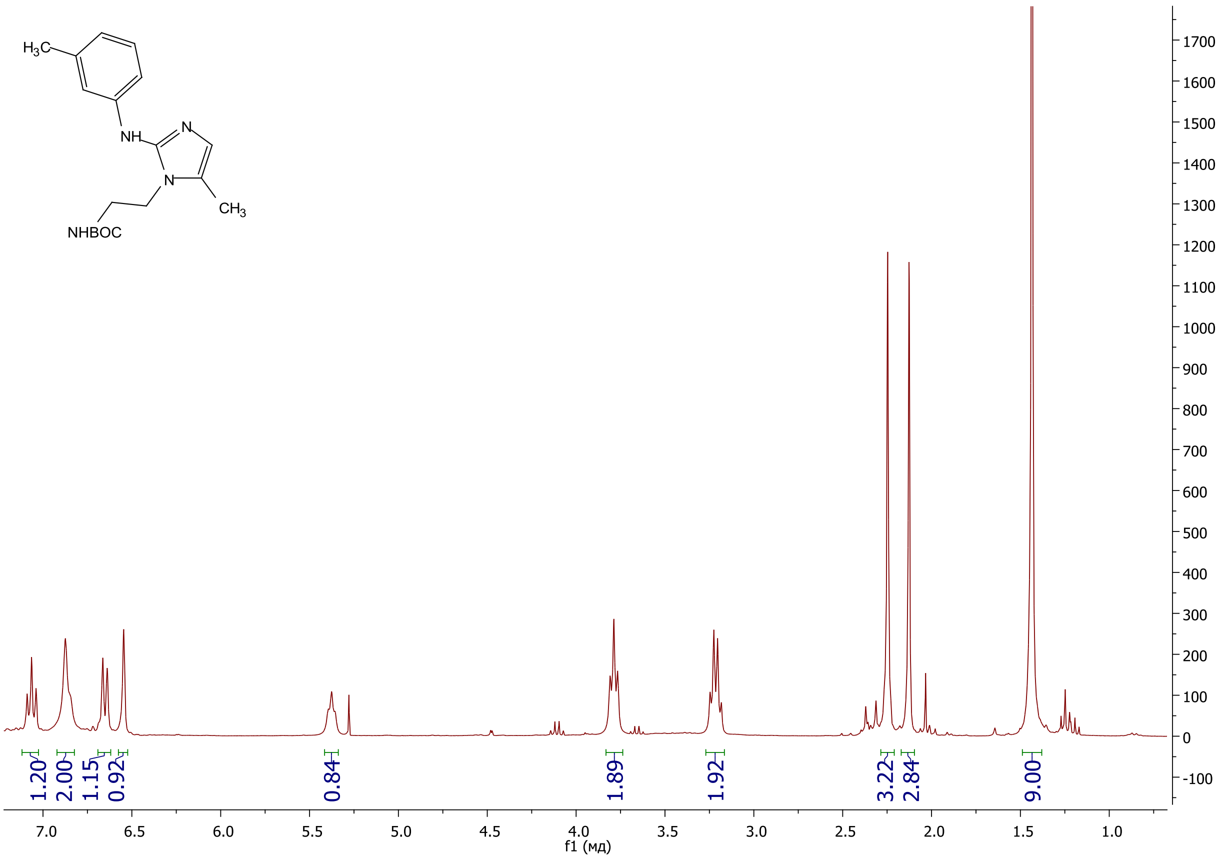

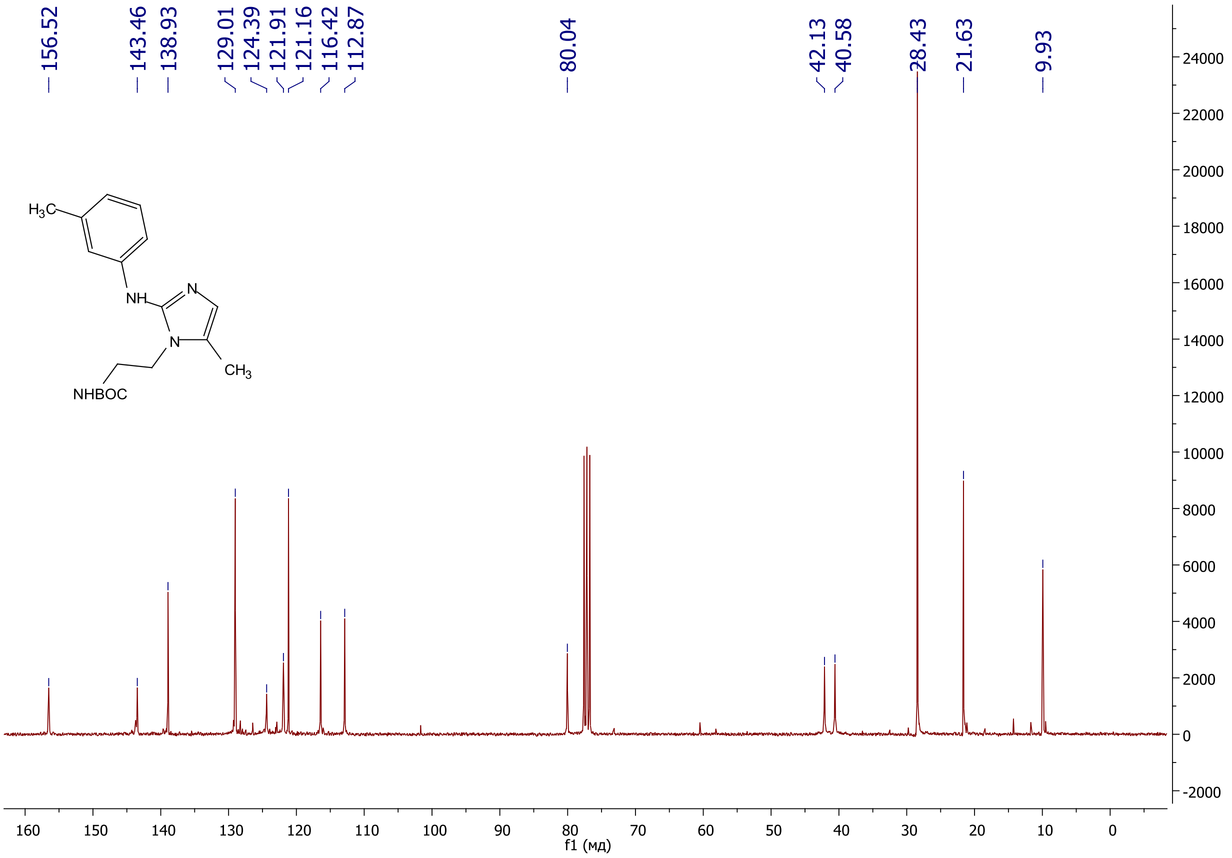

^{1}H and ^{13}C NMR spectra for compound **5j**

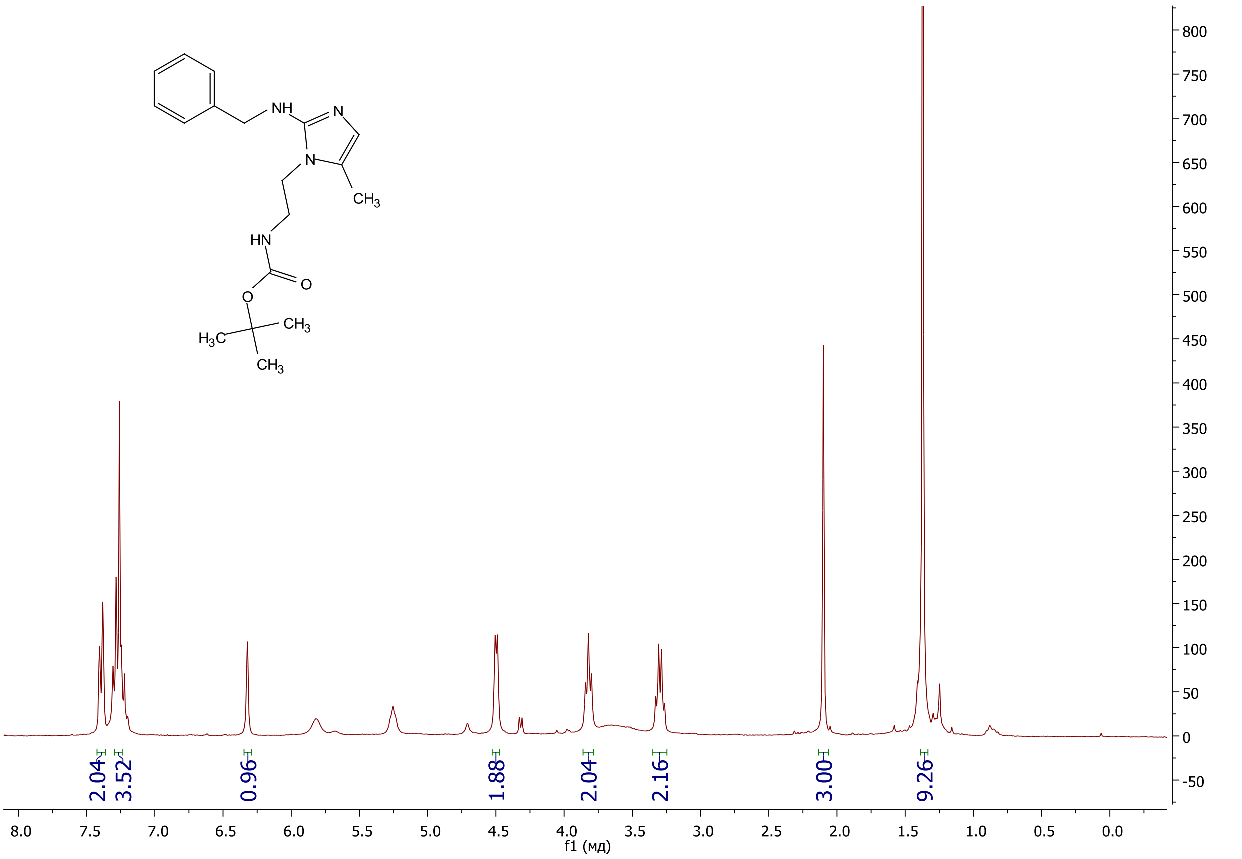

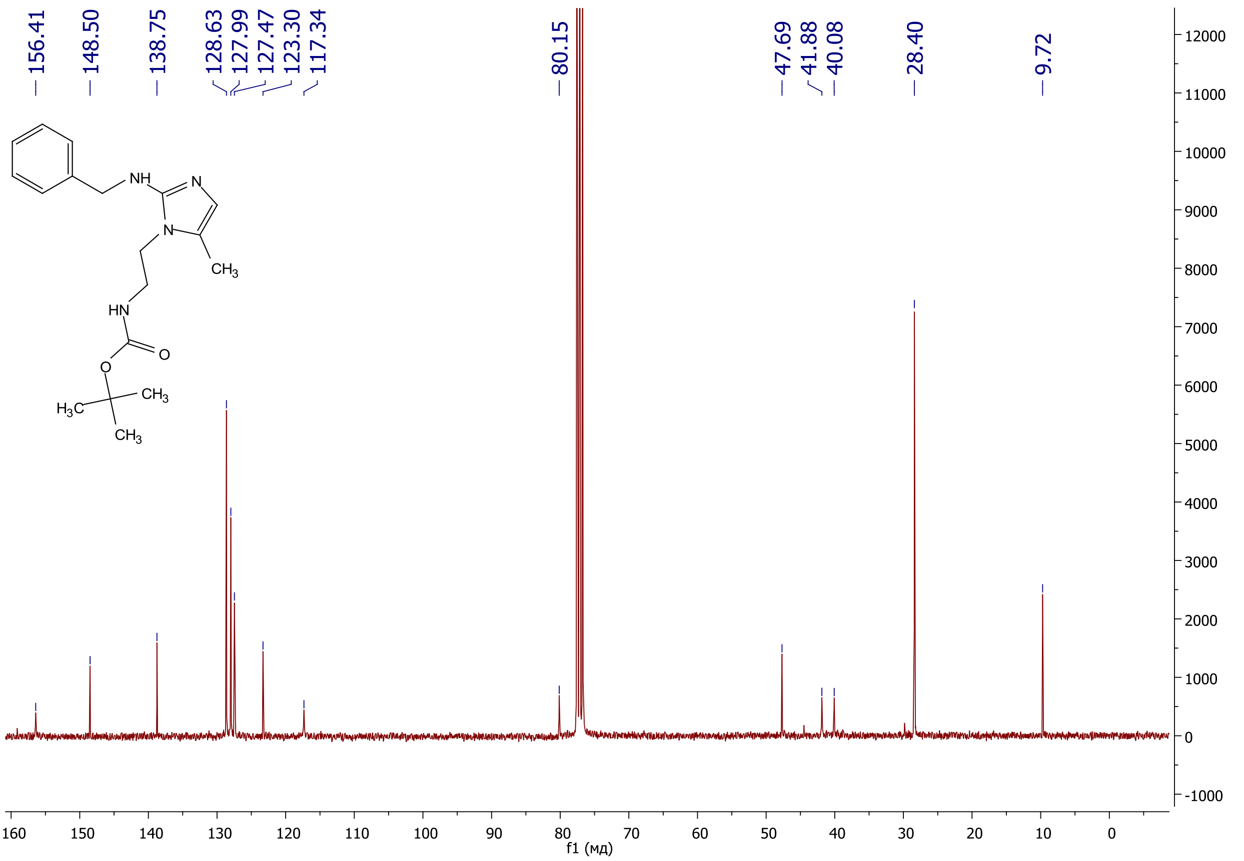

^{1}H and ^{13}C NMR spectra for compound **5k**

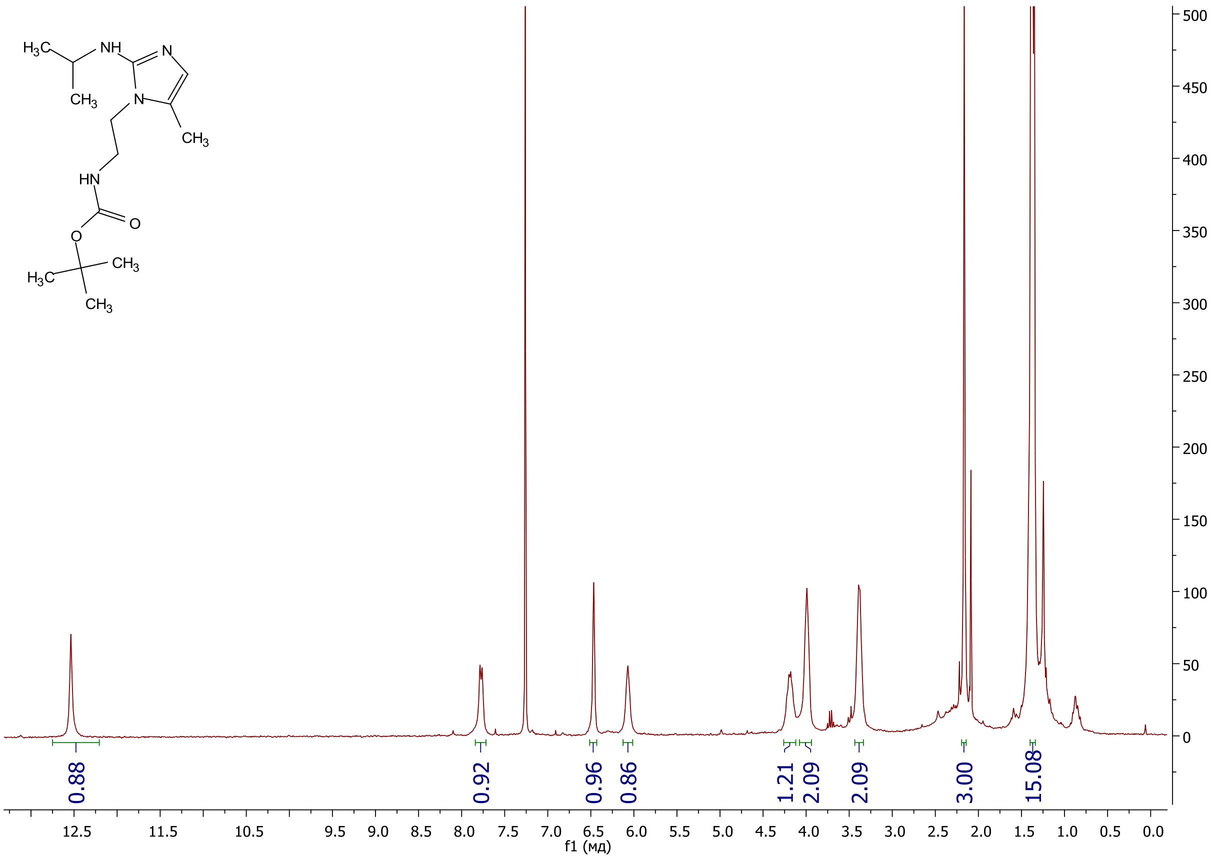

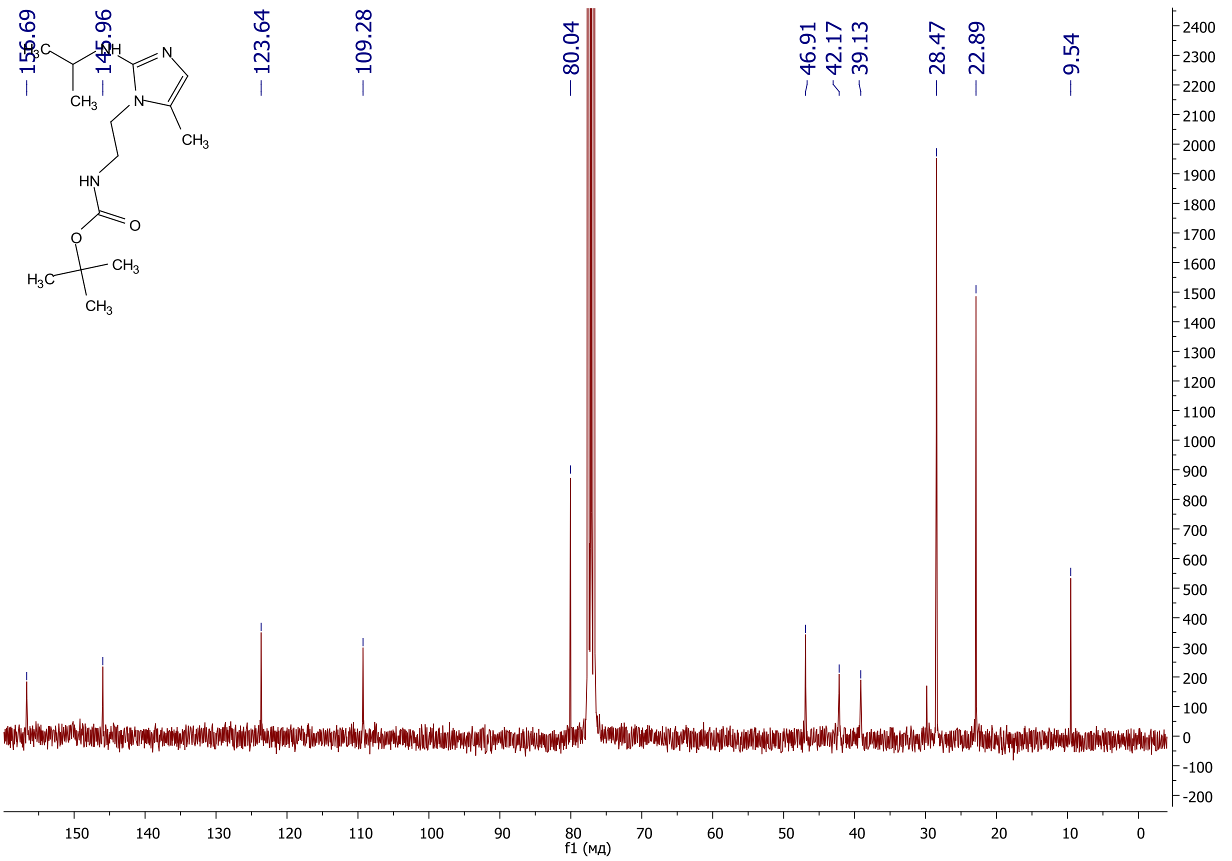

^{1}H and ^{13}C NMR spectra for compound **5l**

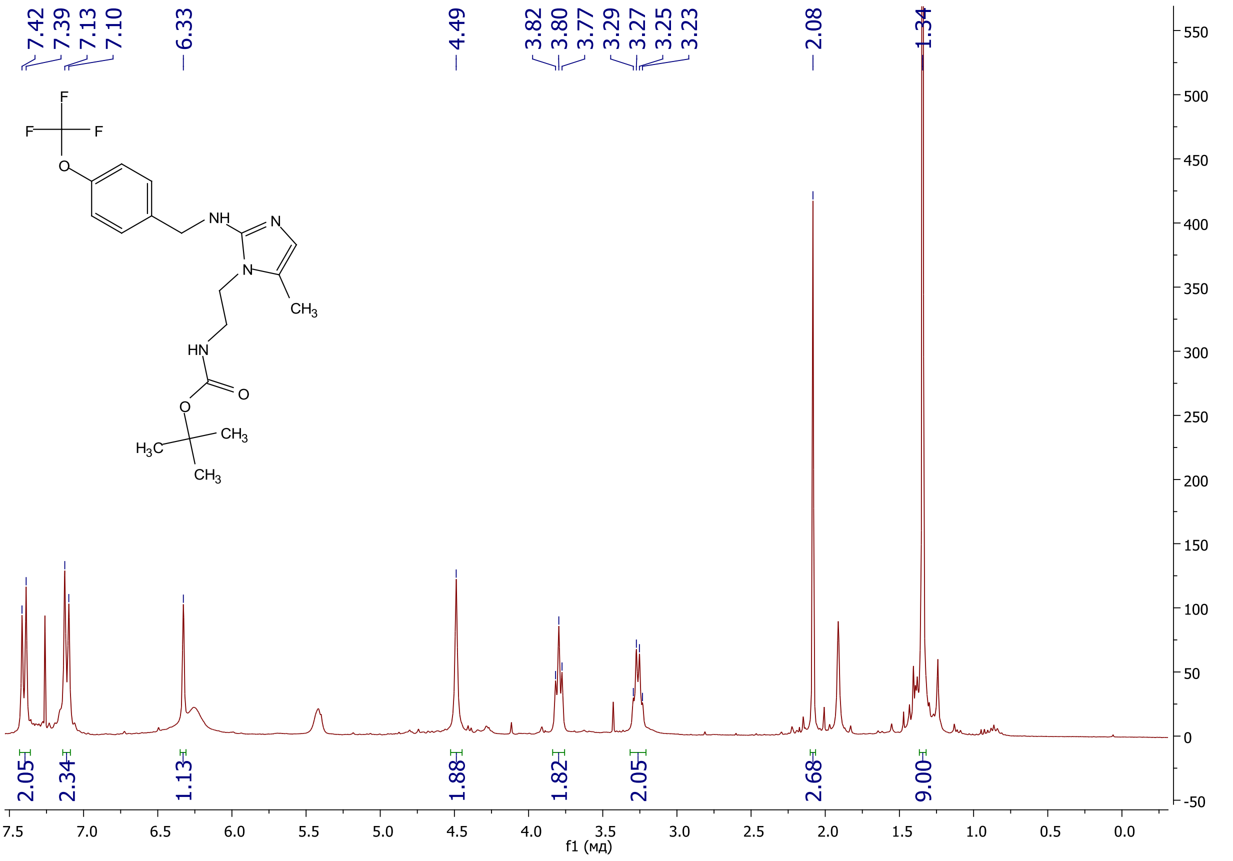

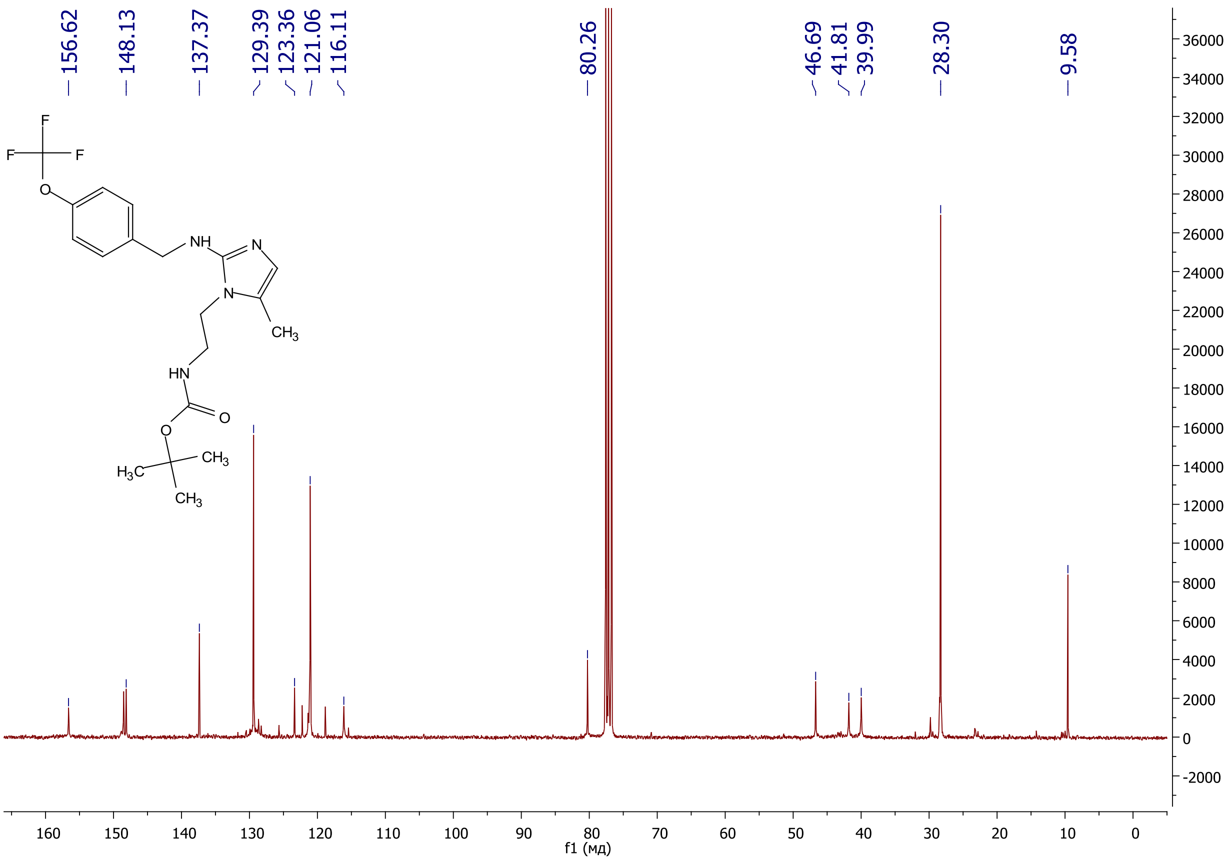

Supplement: File 1 — General experimental information, synthetic procedures, analytical data and NMR spectra for the reported compounds. [file Beilstein_J_Org_Chem-15-1061-s001.pdf]
